# Characterization of mortality and high-risk characteristics of thyroid cancer in Filipinos using the California Cancer Registry

**DOI:** 10.3389/fpubh.2022.1104607

**Published:** 2023-01-19

**Authors:** Robert Hsu, Kai-Ya Tsai, Krithika Chennapan, Katherine Y. Wojcik, Alice W. Lee, Jorge J. Nieva, Lihua Liu

**Affiliations:** ^1^Division of Medical Oncology, Department of Internal Medicine, Keck School of Medicine, University of Southern California, Los Angeles, CA, United States; ^2^Norris Comprehensive Cancer Center, Keck School of Medicine, University of Southern California, Los Angeles, CA, United States; ^3^Department of Population and Public Health Sciences, Keck School of Medicine, University of Southern California, Los Angeles, CA, United States; ^4^Los Angeles Cancer Surveillance Program, Los Angeles, CA, United States; ^5^Department of Internal Medicine, Keck School of Medicine, University of Southern California, Los Angeles, CA, United States; ^6^Department of Public Health, California State University, Fullerton, Fullerton, CA, United States

**Keywords:** thyroid cancer, Filipinos, Asian Pacific Islander, racial/ethnic disparities, California Cancer Registry (CCR)

## Abstract

**Introduction:**

Filipinos are the third largest Asian American subgroup and have the highest incidence of thyroid cancer among all races. To better understand this racial/ethnic disparity in thyroid cancer affecting Filipinos we analyzed the California Cancer Registry (CCR) data in Filipino thyroid cancer cases from 1988 to 2018.

**Methods:**

97,948 thyroid cancer cases in California from 1988 to 2018 (until 2015 for Asian subgroups) were evaluated. We examined the case distribution by sex, age at diagnosis, race/ethnicity including Asian ethnic subgroups, histology, TNM stage, tumor size, lymph node involvement, lymphovascular invasion, and multifocality. We also looked at treatment data including surgery and radiation including radioactive iodine therapy. We calculated age-adjusted mortality rates (AAMR) for each major racial group and each Asian ethnic subgroup. Binary logistic regression was used to determine the likelihood of high-risk characteristics and treatment when comparing Filipinos to other racial/ethnic groups. Kaplan-Meier Estimate was performed to evaluate thyroid cancer survival across all race/ethnicities. Multivariate Cox proportion hazards regression was performed to evaluate mortality risk from all causes of death by race.

**Results:**

There were 5,243 (5.35%) Filipino thyroid cancer cases in California from 1988 to 2018. Filipinos had the highest AAMR (1.22 deaths per 100,000) in 2015. Filipinos had a higher likelihood of Stage IV thyroid cancer compared with Non-Hispanic Whites, Non-Hispanic Blacks, Hispanics and nearly all Asian subgroups. Filipinos had a worse 5-year and 10-year overall survival (OS) than the combination of all other Asian/Pacific Islanders. Filipinos compared to Non-Hispanic Whites had significant mortality risk in overall and papillary thyroid cancer cases (Overall HR: 1.10, 95% CI 1.07–1.13, *p* < 0.0001, Papillary HR: 1.11, 95% CI 1.07–1.14, *p* < 0.0001) when adjusted for race/ethnicity, age, gender, socioeconomic status, and stage. When stratified by Charlson comorbidity score, Filipinos compared to Non-Hispanic Whites still had significant mortality risk (Charlson 0 HR: 1.07, 95% CI 1.02–1.11, *p* = 0.0017, Charlson 1+ HR: 1.07 95% CI 1.002–1.14, *p* = 0.0434).

**Conclusions:**

Filipino thyroid cancer patients have higher incidences of high-risk pathological features and greater AAMR and mortality risk. These findings warrant further investigation into better understanding the connection between the greater incidence of high-risk characteristics and increased mortality in Filipinos.

## Introduction

The incidence of thyroid cancer has continued to rise over the past 15 years across all races, yet Filipinos remain disproportionately affected (1). Previous research studies on a retrospective single center level have suggested that Filipinos have greater prevalence of high-risk characteristics for advanced thyroid cancer including lymph node metastasis, multifocality, tumors>1 cm, and age < 45 years old (2–6). There have not been specific cancer registry studies looking at specific high-risk features and mortality in Filipinos previously in the literature though Filipinos have been shown to have high incidence of thyroid cancer compared to other racial/ethnic groups (7). However, SEER data has shown cervical lymph node metastasis in young thyroid cancer patients showed significantly worsened 10-year OS while another study using SEER data showed that mortality risk from Stage IVB papillary thyroid cancer increased in patients particularly those with tumor sizes >4 cm (5, 6, 8). Previous studies on thyroid cancer in Filipinos have also shown higher age-adjusted mortality compared to Non-Filipino Asian and Non-Hispanic Whites, but studies with more inclusive Asian Pacific Islander subgroup comparisons are not available (9–11). This has shown to be important as our group recently evaluated thyroid cancer incidence across different Asian Pacific Islander subgroups and showed significant differences in these subgroups, as Filipinos had the highest age adjusted incidence rates while a significantly lower incidence of medullary thyroid cancers (12).

Many of the hypotheses for these persistent disparities in Filipinos have been attributed to occupational exposures particularly radiation exposure. A cancer surveillance program at the Los Angeles County/University of Southern California Medical Center showed increased thyroid cancer incidence in Filipinos who had higher educational attainment particularly in healthcare, notably male radiologic technicians, and dentists, and both male and female physicians (10). Many Filipinos who immigrated to the U.S. in the 1970's−1980's were nurses and Filipinos still constitute 75% of all foreign nurses in the U.S. nurse workforce (9). Previous exposure to ionized radiation has been shown to lead to higher percentages of multifocal disease, extrathyroidal spread, stage IV disease, and death in thyroid cancer, but multiple studies have suggested otherwise including studies on healthcare workers (13, 14).

Our study seeks to evaluate incidences of high-risk pathological features in advanced thyroid cancer including Stage IV at diagnosis, positive lymph node involvement, lymphovascular invasion, and multifocality while also evaluating thyroid cancer survival and mortality in Filipinos using population-based cancer registry data. The California Cancer Registry (CCR) represents a valuable resource for better understanding the demographics of thyroid cancer in the Filipino community because California has the largest Asian population in the United States (15). The aim of this study is to help better characterize the pathology of thyroid cancer in Filipinos alongside other races and Asian ethnicities while also examining mortality and survival in Filipinos at a cancer registry level.

## Methods

### Data source/study population

This records-based study included 97,948 thyroid cancer cases diagnosed in 1988–2018 from the California Cancer Registry (CCR) using the CCR Research File of December 2020 in SEER^*^Stat readable format. Demographic characteristics consisted of sex (male, female), age at diagnosis (0–39, 40–49, 50–59, 60–69, 70+ years) and race/ethnicity, which includes Asian-Pacific Islander, Non-Hispanic Whites, Non-Hispanic Blacks, and Hispanics. The Asian-Pacific Islander category was further disaggregated into the following subgroups: Filipino, Chinese, Japanese, Korean, Vietnamese, South Asian (which includes Asian Indian, Pakistani, NOS, Other South Asian, Bangladeshi, Bhutanese, Nepalese, Sikkimese, and Sri Lankan), and Other Southeast Asians (which includes Laotian, Hmong, Cambodian, and Thai). Due to small sample sizes, American Indian and Hawaiian/Pacific Islander groups (e.g., Guamanians/Chamorros, Samoans) were excluded from the analysis.

Cases of thyroid cancer were identified using *International Classification of Disease for Oncology*, Third Edition (ICD-O-3) with site code C73.9– Thyroid gland. Histotypes included follicular (ICD-O-3 codes 8290, 8330, 8331, 8332, 8333, 8335, 8337, 8339, 8346), papillary (ICD-O-3 codes 8050, 8260, 8261, 8262, 8263, 8340, 8341, 8342, 8343, 8344, 8347), medullary (ICD-O-3 codes 8345, 8510, 8346, 8347), and anaplastic (ICD-O-3 codes 8020, 8021, 8022).

We studied tumor staging by considering overall TNM stage (Stage I-IV) along with specific tumor stage (T0, T1, T2, T3, T4), nodal stage (N0, N1), and metastasis (M0, M1) in cases from 2004 to 2018, using AJCC staging 6th−8th editions. In addition, the registry recorded lymphovascular invasion, which consisted of “lymphovascular invasion present,” “lymphatic and small vessel invasion only,” “venous invasion only,” and “both lymphatic and small vessel and venous invasion” was only collected from 2010–2018. Multifocality was noted in cases from 1988 to 2017. Information on surgery (no surgery, lobectomy/local surgery, subtotal or near total thyroidectomy, total thyroidectomy, thyroidectomy/surgery, NOS, and unknown) was available starting in 2003 and radiation (no radiation, isotopes, radiation/combination/other, and unknown) from 1988 to 2017, which was when information on radiation was last available.

### Statistical analysis

Age-adjusted mortality rates (AAMR) and Average Annual Percent Change (AAPC) for mortality for the time period 1988–2018 was calculated for Non-Hispanic White, Non-Hispanic Black, and Asian/Pacific Islander groups. For Asian subgroups, data was available from 1988 to 2015.

Binary logistic regression was used to determine likelihood of certain histology and high-risk pathologic characteristics such as Stage IV disease, T4 tumor stage, N1 node stage, lymphovascular invasion, multifocality, total thyroidectomy, and radiation including radioactive iodine (RAI) between Filipinos and other race/ethnicities, which served as the referent group. Kaplan-Meier analysis was performed to estimate thyroid cancer survival at 1- year, 5 -years, and 10- years for all races and by Asian subgroups. For the Asian Pacific Islander race for survival analysis, Filipinos were separated from the rest of the Asian Pacific Islander race.

To evaluate mortality risk from all causes of death by race/ethnicity in both thyroid cancer cases overall and papillary thyroid cancer cases, multivariate hazard cox ratio regression analysis was performed adjusting for race/ethnicity (Non-Hispanic Whites, Hispanics, Non-Hispanic Black, Filipino, Asian/Pacific Islander excluding Filipino), sex (female, male), age (0–39, 40–64, 65+), socioeconomic status (lowest, lower-middle, middle, upper-middle, highest), and SEER summary stage (localized, regional, distant, unknown, *in situ*). To evaluate whether these mortality risks maybe attributed to thyroid cancer vs. other comorbidities, we stratified by Charlson comorbidity score (Charlson score 0 and Charlson score 1 or greater).

Tests for statistical significance were two-sided and considered statistically significant at *p* < 0.05. Statistical analysis was performed using SAS software, release 9.4 (SAS Institute, Cary, NC).

## Results

### Demographics

There were a total of 97,948 total thyroid cancer cases from 1988 to 2018; of these, 52,425 Non-Hispanic White, 24,928 Hispanic, 3,680 Non-Hispanic Black, 15,421 Asian/Pacific Islander [including Filipinos (*n* = 5,243), Chinese (*n* = 3,390), Vietnamese *n* = 1,664), Koreans (*n* = 1,327), Japanese (*n* = 760), Other Southeast Asians (*n* = 136)], and 1,494 unknown. Similar to all race/ethnicities, Filipinos had a high female predominance (80.11%) (Table 1). There was a fairly even distribution of age at diagnosis in Filipinos with 63.4% of Filipino thyroid cases being diagnosed between the ages of 40–69 (Table 1A).

**Table 1:** Patient/tumor characteristics by race/ethnicity.

	**Non-Hispanic White**	**Non-Hispanic Black**	**Hispanic**	**Asian Pacific Islander**	**Filipino**	**Chinese**	**Japanese**	**Korean**	**Vietnamese**	**South Asian**	**Other Southeast Asians**	**Unknown**
Total: 97,948* (including 9 cases with missing sex info)	52,425 (53.52)	3,680 (3.76)	24,928 (25.45)	15,421 (15.74)	**5,243 (5.35)**	3,390 (3.46)	760 (0.78)	1,327 (1.35)	1,664 (1.70)	1,170 (1.19)	436 (0.45)	1,494 (1.53)
Male (Total: 24,017)	14,778 (28.19)	861 (23.40)	4,768 (19.13)	3,274 (21.23)	**1043 (19.89)**	756 (22.30)	169 (22.24)	308 (23.21)	344 (20.67)	298 (25.47)	84 (19.27)	336 (22.49)
Female (Total: 73,922)	37,641 (71.80)	2,819 (76.60)	20,157 (80.86)	12,147 (78.77)	**4,200 (80.11)**	2,634 (77.70)	591 (77.76)	1,019 (76.79)	1,320 (79.33)	872 (74.53)	352 (80.73)	1,158 (77.51)
**A. Age at diagnosis from 1988-2018 (2015 in Asian subgroups)**												
Total: 97,948	52,425 (53.52)	3,680 (3.76)	24,928 (25.45)	15,421 (15.74)	**5,243 (5.35)**	3,390 (3.46)	760 (0.78)	1,327 (1.35)	16,64 (1.70)	1,170 (1.19)	436 (0.45)	1,494 (1.53)
< 40 years	13,909 (26.53)	862 (23.42)	9,652 (38.72)	4,492 (29.12)	**1,190 (22.70)**	1,023 (30.18)	167 (21.97)	319 (24.04)	538 (32.33)	589 (50.34)	133 (30.50)	536 (35.88)
40–49 years	10,762 (20.53)	791 (21.49)	5,590 (22.42)	3,421 (22.18)	**1,128 (21.51)**	770 (22.71)	148 (19.47)	284 (21.40)	403 (24.22)	258 (22.05)	91 (20.87)	337 (22.56)
50–59 years	11,183 (21.33)	871 (23.67)	4,651 (18.66)	3,165 (20.52)	**1,177 (22.45)**	655 (19.32)	145 (19.08)	304 (22.91)	337 (20.25)	154 (13.16)	93 (21.33)	330 (22.09)
60–69 years	8,886 (16.95)	641 (17.42)	2,932 (11.76)	2,475 (16.05)	**1,019 (19.44)**	524 (15.46)	149 (19.61)	230 (17.33)	218 (13.10)	103 (8.80)	70 (16.06)	179 (11.98)
70+ years	7,685 (14.66)	515 (13.99)	2,103 (8.44)	1,868 (12.11)	**729 (13.90)**	418 (12.33)	151 (19.87)	190 (14.32)	168 (10.10)	66 (5.64)	49 (11.24)	112 (7.50)
**B. Histology from 1988-2018 (2015 in Asian subgroups)**												
Total: 97,948* (including 2,424 cases with missing histology info)	52,425 (53.52)	3,680 (3.76)	24,928 (25.45)	15,421 (15.74)	**5,243 (5.35)**	3,390 (3.46)	760 (0.78)	1,327 (1.35)	1,664 (1.70)	1,170 (1.19)	436 (0.45)	1,494 (1.53)
Papillary	44,528 (84.94)	2,887 (78.45)	21,968 (88.13)	13,677 (88.70)	**4,640 (88.50)**	3,025 (89.23)	671 (88.29)	1,224 (92.24)	1,451 (87.20)	1,032 (87.20)	347 (79.59)	1,291 (86.35)
Follicular	4,777 (9.11)	520 (14.13)	1,709 (6.86)	1,053 (6.83)	**367 (7.00)**	213 (6.28)	52 (6.84)	54 (4.07)	148 (8.89)	80 (6.84)	45 (10.32)	119 (7.96)
Medullary	1,208 (2.30)	90 (2.45)	486 (1.95)	167 (1.08)	**40 (0.76)**	45 (1.33)	-	14 (1.06)	18 (1.08)	26 (2.22)	-	27 (1.81)
Anaplastic	575 (1.10)	47 (1.08)	222 (0.89)	167 (1.08)	**68 (1.30)**	31 (0.91)	-	12 (0.90)	15 (0.90)	-	-	-
Missing	1,337 (2.55)	136 (3.70)	543 (2.18)	357 (2.31)	**128 (2.44)**	76 (2.24)	24 (3.16)	23 (1.73)	32 (1.92)	-	29 (6.65)	-
**C. Stage at diagnosis from 2004-2018 (2015 in Asian subgroups)**												
Total: 67,782	34,031 (50.21)	2,576 (3.80)	18,702 (27.59)	11,300 (16.67)	**3,712 (5.48)**	2,543 (3.75)	458 (0.68)	998 (1.47)	1,157 (1.71)	965 (1.42)	299 (0.44)	1,173 (1.73)
I	21,548 (63.32)	1,572 (61.02)	12,433 (66.48)	7,005 (61.99)	**2,094 (56.41)**	1,607 (63.19)	251 (54.80)	576 (57.72)	748 (64.65)	745 (77.20)	179 (59.87)	756 (64.45)
II	2,973 (8.74)	270 (10.48)	1,196 (6.40)	784 (5.08)	**299 (8.05)**	167 (6.57)	31 (6.77)	55 (5.51)	94 (8.12)	44 (4.56)	22 (7.36)	65 (5.54)
III	4,127 (12.13)	288 (11.18)	2,094 (11.20)	1,493 (13.21)	**540 (14.55)**	340 (13.37)	79 (17.25)	172 (17.23)	147 (12.71)	62 (6.42)	39 (13.04)	91 (7.76)
IV	3,176 (9.33)	243 (9.43)	1,791 (9.58)	1,254 (11.10)	**494 (13.31)**	264 (10.38)	72 (15.72)	107 (10.72)	93 (8.04)	78 (8.08)	39 (13.04)	50 (4.26)
Unknown	2,207 (6.49)	203 (7.88)	1,188 (6.35)	764 (6.76)	**285 (7.68)**	165 (6.49)	25 (5.46)	88 (8.82)	75 (6.48)	36 (3.73)	20 (6.69)	211 (17.99)
**D. Tumor stage from 2004-2018 (2015 in Asian subgroups)**												
Total: 67,782* (including 420 cases with missing tumor stage info)	34,031 (50.21)	2,576 (3.80)	18,702 (27.59)	11,300 (16.67)	**3,712 (5.48)**	2,543 (3.75)	458 (0.68)	998 (1.47)	1,157 (1.71)	965 (1.42)	299 (0.44)	1,173 (1.73)
T0/T1	18,961 (55.72)	1,328 (51.55)	8,625 (46.12)	5,539 (49.02)	**1,723 (46.42)**	1,315 (51.71)	235 (51.31)	483 (48.40)	416 (49.06)	498 (51.61)	128 (42.81)	557 (47.49)
T2	5,765 (16.94)	453 (17.59)	3,278 (17.53)	1,880 (16.64)	**647 (17.43)**	420 (16.52)	58 (12.66)	123 (12.32)	138 (16.27)	185 (19.17)	56 (18.73)	177 (15.09)
T3	6,093 (17.90)	500 (19.41)	4,496 (24.04)	2,558 (22.64)	**860 (23.17)**	555 (21.82)	102 (22.27)	248 (24.85)	204 (24.06)	196 (20.31)	72 (24.08)	186 (15.86)
T4	1,614 (4.74)	128 (4.97)	1,317 (7.04)	738 (6.53)	**290 (7.81)**	135 (5.31)	47 (10.26)	73 (7.31)	47 (5.54)	50 (5.18)	16 (5.35)	34 (2.90)
Tx	1,339 (45.72)	138 (5.36)	797 (4.26)	797 (4.26)	**160 (4.31)**	101 (3.97)	13 (2.84)	65 (6.51)	53 (5.07)	31 (3.21)	20 (6.69)	170 (14.49)
NA	92 (0.27)	-	53 (0.28)	53 (0.28)	-	-	-	-	-	-	-	12 (1.02)
Missing	167 (0.49)	-	136 (0.73)	70 (0.62)	-	-	-	-	-	-	-	37 (3.15)
**E. Nodal stage from 2004-2018 (2015 in Asian subgroups)**												
Total: 67,782* (including 341 cases with missing nodal stage info)	34,031 (50.21)	2,576 (3.80)	18,702 (27.59)	11,300 (16.67)	**3,712 (5.48)**	2,543(3.75)	458 (0.68)	998 (1.47)	1,157 (1.71)	965 (1.42)	299 (0.44)	1,173 (1.73)
N0	24,318 (71.46)	2,028 (78.73)	8,821 (65.02)	7,401 (65.50)	**2,414 (65.03)**	1,683 (66.18)	298 (65.07)	629 (63.03)	793 (68.54)	620 (64.25)	199 (66.56)	709 (60.44)
N1	8,178 (24.03)	361 (14.01)	4,277 (31.53)	3,299 (29.19)	**1,095 (29.50)**	727 (28.59)	141 (30.79)	302 (30.26)	301 (26.02)	312 (32.33)	81 (27.09)	238 (20.29)
NA	85 (0.25)	-	52 (0.28)	30 (0.27)	-	-	-	-	-	-	-	12 (1.02)
Unknown	1,320 (3.88)	161 (6.25)	906 (4.84)	513 (4.54)	**175 (4.71)**	118 (4.64)	17 (3.71)	62 (6.21)	51 (4.41)	29 (3.01)	14 (4.68)	177 (15.09)
Missing	130 (0.38)	-	110 (0.59)	57 (0.50)	-	-	-	-	-	-	-	37 (3.15)
**F. Lymphovascular invasion from 2010-2018 (2015 in Asian subgroups)**												
Total: 46,325* (including 1,814 cases with missing lymphovascular invasion info)	22,367 (48.28)	1,730 (3.73)	13,392 (28.91)	7,953 (17.17)	**2,527 (5.45)**	1,796 (3.88)	289 (0.62)	710 (1.53)	787 (1.70)	742 (1.60)	214 (0.46)	883 (1.91)
Yes	2,831 (12.66)	193 (11.16)	2,154 (16.08)	1,122 (14.11)	**407 (16.11)**	221 (12.31)	37 (12.80)	88 (12.39)	119 (15.12)	100 (13.48)	28 (13.08)	85 (9.63)
No	13,488 (60.30)	925 (53.47)	7,128 (53.23)	4,471 (56.22)	**1,349 (53.38)**	1,043 (58.07)	169 (58.48)	373 (52.54)	455 (57.81)	461 (62.13)	113 (52.80)	441 (49.94)
NA	2,621 (11.72)	330 (19.08)	1,710 (12.77)	986 (12.80)	**355 (12.11)**	204 (11.36)	-	91 (12.82)	87 (11.05)	69 (9.30)	35 (16.36)	113 (12.80)
Unknown	2,686 (12.01)	222 (12.83)	1,792 (13.38)	1,038 (13.05)	**323 (12.78)**	240 (13.36)	40 (13.84)	107 (15.07)	100 (12.71)	83 (11.19)	-	175 (19.82)
Missing	741 (3.31)	60 (3.47)	608 (4.54)	336 (4.22)	**93 (3.68)**	88 (4.90)	-	51 (7.18)	26 (3.30)	29 (3.91)	-	69 (7.81)
**G. Multifocality from 1988-2017 (2015 in Asian subgroups)**												
Total: 97,948	52,425 (53.52)	3,680 (3.76)	24,928 (25.45)	15,421 (15.74)	**5,243 (5.35)**	3,390 (3.46)	760 (0.78)	1,327 (1.35)	1,664 (1.70)	1,170 (1.19)	436 (0.45)	1,494 (1.53)
Solitary	18,200 (34.72)	1,510 (41.03)	9,503 (38.12)	5,625 (36.48)	**1,809 (34.50)**	1,313 (38.73)	219 (28.82)	500 (37.68)	610 (36.66)	470 (40.17)	147 (33.72)	558 (37.32)
Mulitfocal	12,341 (23.54)	745 (20.24)	6,822 (27.37)	4,254 (27.59)	**1,460 (27.85)**	933 (27.52)	185 (24.34)	383 (28.86)	397 (23.86)	360 (30.77)	112 (25.69)	346 (23.14)
No evidence of thyroid	128 (0.24)	15 (0.41)	73 (0.29)	39 (0.25)	**12 (0.23)**	-	-	-	-	-	-	-
Unknown	956 (1.82)	90 (2.45)	547 (2.19)	370 (2.40)	**125 (2.38)**	-	-	-	-	-	-	-
Missing/NA	20,800 (39.68)	1,320 (35.87)	7,983 (32.02)	5,133 (33.28)	**1,837 (35.04)**	1,058 (31.21)	340 (44.74)	397 (29.92)	614 (36.90)	313 (26.75)	160 (36.70)	473 (31.71)

-, By CCR regulations, any case count smaller than 11 must be withheld to prevent potential patient identification. Bolded values signify values of Filipino ethnicity.

### Disease characteristics

**Figure 1:**
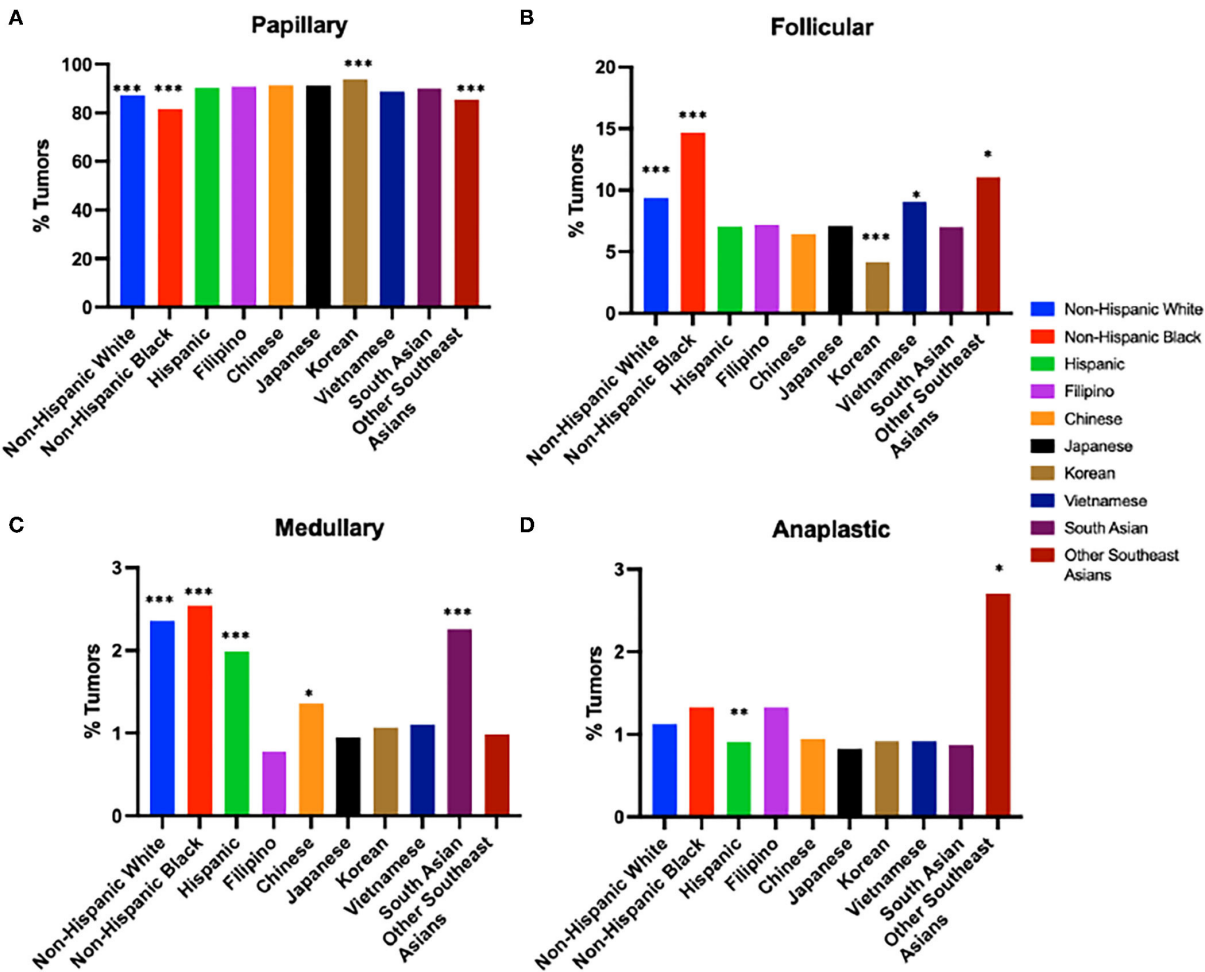
Tumor histology by race/ethnicity from 1988 to 2018 (2015 in Asian subgroups). **(A)** Papillary Thyroid Cancer. **(B)** Follicular Thyroid Cancer. **(C)** Medullary Thyroid Cancer. **(D)** Anaplastic Thyroid Cancer. Odds ratio for prevalence compared to Filipinos. ***p-value < 0.0005, **p-value < 0.005, *p-value < 0.05.

Papillary subtype was by far the most prevalent in all races, accounting for 85% of total cases. This was followed by 8% follicular, 2% medullary, and 1% anaplastic (Figure 1A, Table 1B). Papillary thyroid carcinoma was also the most common histotype in Filipinos, accounting for 89% of thyroid cancer cases (Figure 1A, Table 1B). Filipinos were significantly more likely than Non-Hispanic Whites (OR: 1.366; 95% CI: 1.250–1.492, *p* < 0.0001), Non-Hispanic Blacks (OR: 2.119; 95% CI 1.886–2.375, *p* < 0.0001), and Other Southeast Asians (OR: 1.984; 95% CI 1.550–2.544, *p* < 0.0001) to have papillary thyroid cancer but less likely than Koreans (OR: 0.652, 95% CI: 0.524–0.812, *p* = 0.0001). Detailed subtype comparisons of Filipinos vs. other race/ethnicities and Asian subgroups are noted in Figures 1A–D, Table 1, and Supplementary Table 1A.

**Figure 2:**
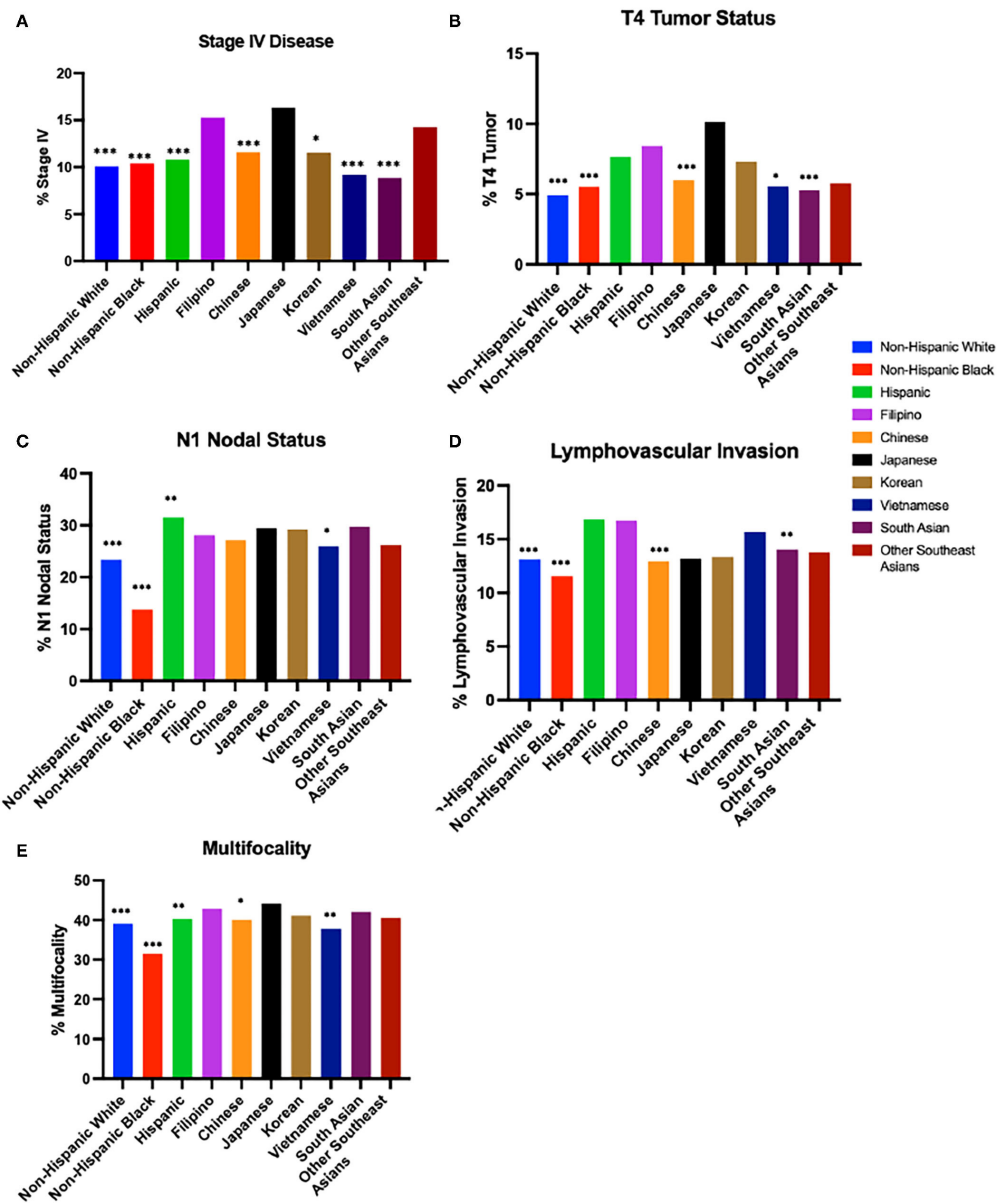
Disease characteristics by race/ethnicity. **(A)** Stage IV Disease at diagnosis from 2004 to 2018 (2015 in Asian subgroups). **(B)** T4 Tumor Status from 2004 to 2018 (2015 in Asian subgroups). **(C)** N1 Nodal Status from 2004 to 2018 (2015 in Asian subgroups). **(D)** Lymphovascular Invasion from 2010 to 2018 (2015 in Asian subgroups). **(E)** Multifocality Odds ratio for prevalence compared to Filipinos from 2004 to 2018 (2015 in Asian subgroups). ***p-value < 0.0005, **p-value < 0.005, *p-value < 0.05.

13.31% of Filipinos had Stage IV disease at diagnosis. Filipinos were more likely to have Stage IV disease compared to Non-Hispanic Whites (OR: 1.52; 95% CI:1.37–1.69; *p* < 0.0001), Non-Hispanic Blacks (OR: 1.49; 95%CI: 1.26–1.75; *p* < 0.0001), Hispanics (OR: 1.48; 95%CI: 1.32–1.65, *p* < 0.0001), and all Asian subgroups with the exception of Japanese (OR: 0.84; 95%CI: 0.64–1.11; *p* = 0.22) and Other Southeast Asians (OR: 1.05; 95%CI:0.73–1.49; *p* = 0.80) (Figure 2A, Table 1C, and Supplementary Table 1B).

Looking specifically at tumor and nodal staging, 7.81% of Filipinos had T4 tumor stage, 29.50% of Filipinos had N1 nodal stage, 16.11% of Filipinos had lymphovascular invasion, and 27.85% of Filipinos presented with multifocal disease. Filipinos were more likely than Non-Hispanic Whites and Non-Hispanic Blacks to have T4 tumor staging (Non-Hispanic Whites OR: 1.464; 95% CI: 1.374–1.559; *p* < 0.0001; Non-Hispanic Blacks OR: 1.258; 95% CI: 1.145–1.383, *p* < 0.0001); N1 nodal stage (Non-Hispanic Whites OR: 1.340; 95% CI: 1.248-1.439, *p* < 0.0001; Non-Hispanic Blacks OR: 1.882; 95% CI: 1.677–2.112, *p* < 0.0001), lymphovascular invasion (Non-Hispanic Whites OR: 1.440; 95% CI: 1.278–1.620, *p* < 0.0001; Non-Hispanic Blacks OR: 1.445; 95% CI: 1.193-1.748, *p* = 0.0002), and multifocal disease (Non-Hispanic Whites OR: 1.190; 95% CI: 1.107–1.280, *p* < 0.0001; Non-Hispanic Blacks OR: 1.642; 95% CI: 1.468-1.834, *p* < 0.0001). Amongst Asian subgroups, Filipinos were more likely than Chinese (OR: 1.232; 95% CI: 1.121–1.354, *p* < 0.0001), Vietnamese (OR: 1.137; 95% CI: 1.005–1.287; *p* = 0.0415), and South Asians (OR: 1.281; 95% CI: 1.121-1.464, *p* = 0.0003) to have T4 tumor staging, more likely than Vietnamese (OR: 1.193; 95% CI: 1.035–1.375, *p* = 0.0148) to have N1 nodal staging, more likely than Chinese (OR: 1.422; 95% CI: 1.185–1.706, *p* = 0.0002) and South Asians (OR: 1.391; 95% CI: 1.091–1.773, *p* = 0.0078) to have lymphovascular invasion, and more likely than Chinese (OR: 1.134, 95% CI: 1.017–1.263, *p* = 0.0236) and Vietnamese (OR: 1.242; 95% CI: 1.074–1.435, *p* = 0.0031) to have multifocal disease. Detailed comparisons of Filipinos vs. other ethnicities and Asian subgroups are noted in Figures 2B–E, Tables 1D–G, and Supplementary Table 1.

### Treatment

**Figure 3:**
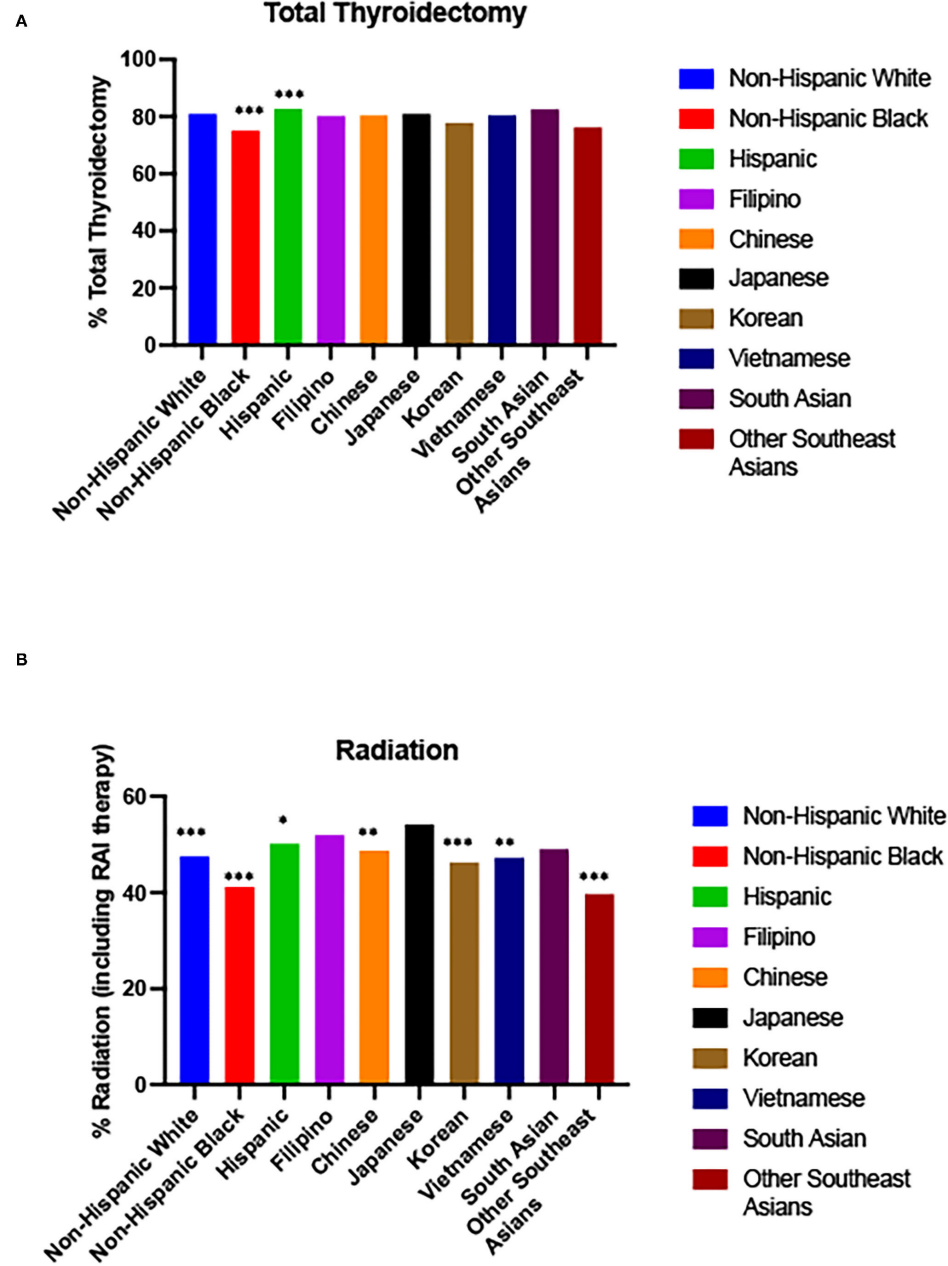
Surgery/radiation by race/ethnicity. **(A)** Total Thyroidectomy from 2003 to 2018 (2015 in Asian subgroups). **(B)** Radiation (including RAI therapy) from 1988 to 2017 (2015 in Asian subgroups). Odds ratio for prevalence compared to Filipinos. ***p-value < 0.0005, ** *p*-value < 0.005, *p-value < 0.05.

76.96% of Filipinos diagnosed with thyroid cancer received a total thyroidectomy. This was significantly increased compared to Non-Hispanic Blacks (OR: 1.230, 95% CI: 1.078–1.403, *p* = 0.0022), Koreans (OR 1.211, 95% CI: 1.009–1.451, *p* = 0.0395), but fewer than Hispanics (OR 0.817 (0.741–0.900), *p* < 0.0001) (Figure 3A, Table 2A, Supplementary Table 2A). 43.59% of Filipinos received isotope therapy and 3.99% received combination therapy. Filipinos were more likely than all race/ethnicities except Japanese and South Asians to receive radiation (including isotopes) (Figure 3B, Table 2B, Supplementary Table 2B).

**Table 2:** Surgery/radiation by race/ethnicity.

	**Non-Hispanic White**	**Non-Hispanic Black**	**Hispanic**	**Asian Pacific Islander**	**Filipino**	**Chinese**	**Japanese**	**Korean**	**Vietnamese**	**South Asian**	**Other Southeast Asians**	**Unknown**
**A. Surgery from 2003-2018**												
Total: 70,488	35,619 (50.53)	2,688 (3.81)	19,311 (27.40)	11,672 (16.56)	**3845 (5.45)**	2,604 (3.69)	473 (0.67)	1,032 (1.46)	1,213 (1.72)	997 (1.41)	314 (0.45)	1,198 (1.70)
No surgery	1,864 (5.23)	170 (6.32)	1,058 (5.48)	760 (6.51)	**250 (6.50)**	171 (6.57)	39 (8.25)	86 (8.33)	58 (4.78)	48 (4.81)	23 (7.32)	214 (17.86)
Lobectomy/local surgery	3,222 (9.05)	315 (11.72)	1,454 (7.53)	1,081 (9.26)	**345 (8.97)**	245 (9.41)	40 (8.46)	101 (9.79)	128 (10.55)	94 (9.43)	39 (12.42)	124 (10.35)
Subtotal or near total thyroidectomy	1,032 (2.90)	109 (4.06)	462 (2.21)	258 (2.21)	**100 (2.60)**	49 (1.88)	-	18 (1.74)	-	-	-	19 (1.59)
Total thyroidectomy	27,398 (76.92)	1,915 (71.24)	15,321 (79.34)	8,979 (76.93)	**2,959 (76.96)**	2,023 (77.69)	366 (77.38)	770 (74.61)	924 (76.17)	793 (79.54)	225 (71.66)	759 (63.36)
Thyroidectomy/surgery, NOS	268 (0.75)	24 (0.89)	176 (0.91)	91 (0.78)	**29 (0.75)**	21 (0.81)	-	14 (1.36)	-	-	-	15 (1.25)
Missing/Unknown	1,835 (5.16)	155 (5.76)	840 (4.35)	503 (4.31)	**162 (4.21)**	95 (3.65)	22 (4.65)	43 (4.17)	67 (5.52)	38 (3.81)	21 (6.69)	67 (5.59)
**B. Radiation from 1988–2017**												
Total: 92,409^*^ (including 189 unknown cases and 2 cases with missing radiation info)	50,019 (54.13)	3,464 (3.75)	23,174 (25.08)	14,408 (15.59)	**4,937 (5.34)**	3,179 (3.44)	722 (0.78)	1,259 (1.36)	1,557 (1.68)	1,062 (1.15)	413 (0.45)	1,344 (1.45)
Isotopes	21,801 (43.59)	1,299 (37.50)	10,735 (46.32)	6,513 (45.20)	**2,340 (47.40)**	1,416 (44.54)	354 (49.03)	540 (42.89)	682 (43.80)	481 (45.29)	148 (35.84)	418 (31.10)
No radiation	26,107 (52.19)	2,024 (58.43)	11,501 (49.63)	7,286 (50.57)	**2,367 (47.94)**	1,624 (51.09)	329 (45.57)	672 (53.38)	819 (52.60)	540 (50.85)	246 (59.56)	888 (66.07)
Radiation/Combination/Other	1,994 (3.99)	128 (3.70)	904 (3.90)	589 (4.09)	**228 (4.62)**	134 (4.22)	38 (5.26)	44 (3.49)	54 (3.47)	40 (3.77)	16 (3.87)	31 (2.31)

-, By CCR regulations, any case count smaller than 11 must be withheld to prevent potential patient identification. Bolded values signify values of Filipino ethnicity.

### Mortality and survival

**Figure 4:**
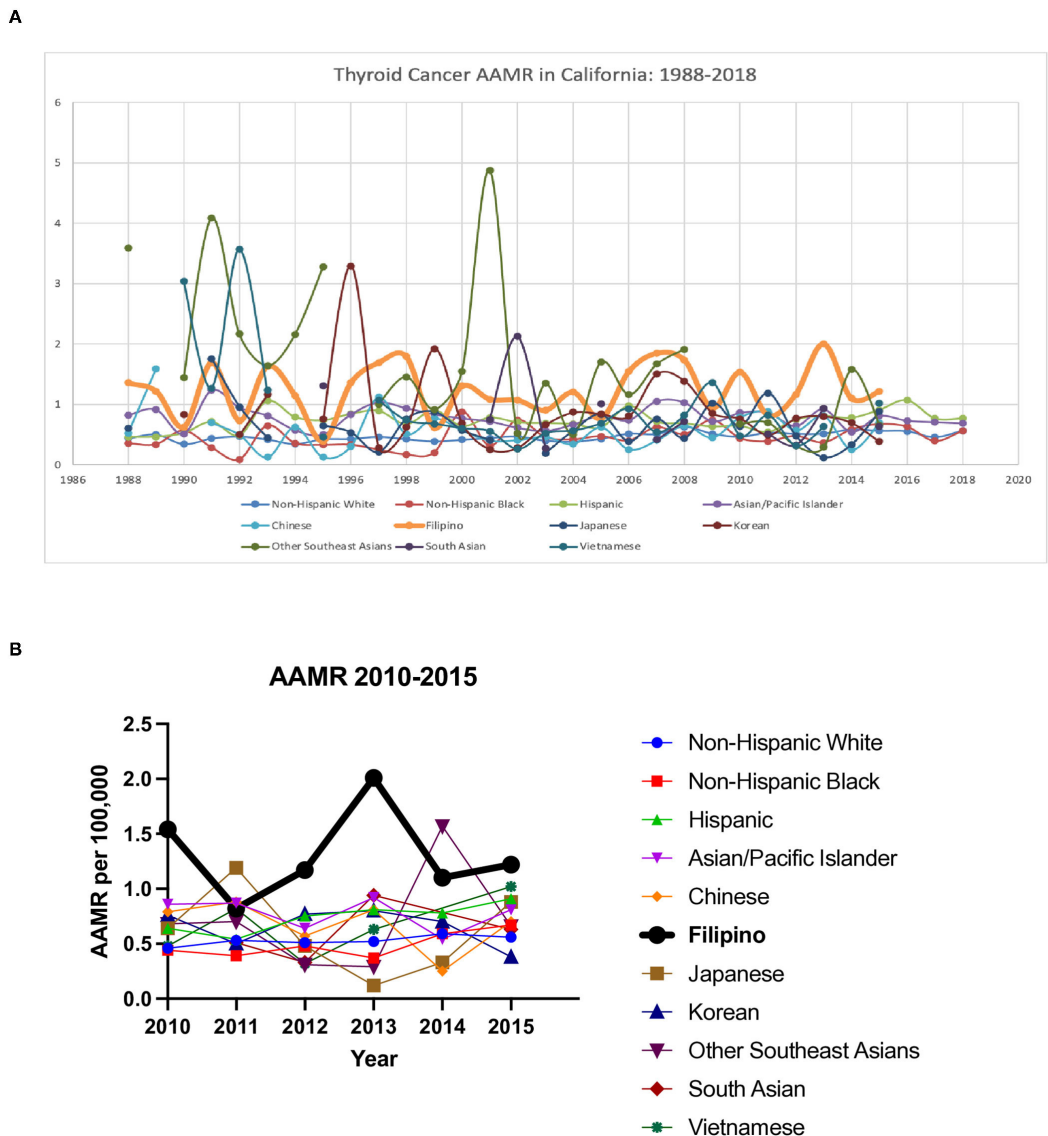
Thyroid cancer age adjusted mortality ratio (AAMR) in California. **(A)** 1988–2018 (2015 in Asian subgroups). **(B)** 2010–2015.

Filipinos had the highest age adjusted mortality rate in 2015 amongst all subgroups (AAMR 1.22 deaths per 100,000), and a general trend of one of the highest or the highest AAMR of all races through the studied time period, including from the most recent 5 years (Figures 4A, B, Supplementary Table 3) Filipinos had a AAPC for mortality of 0.8% (95% CI−1.1-2.7) total and 0.8% in males (95% CI −2–3.6) and −0.7% in females (95% CI −2.9–1.5) (Supplementary Table 4).

**Figure 5:**
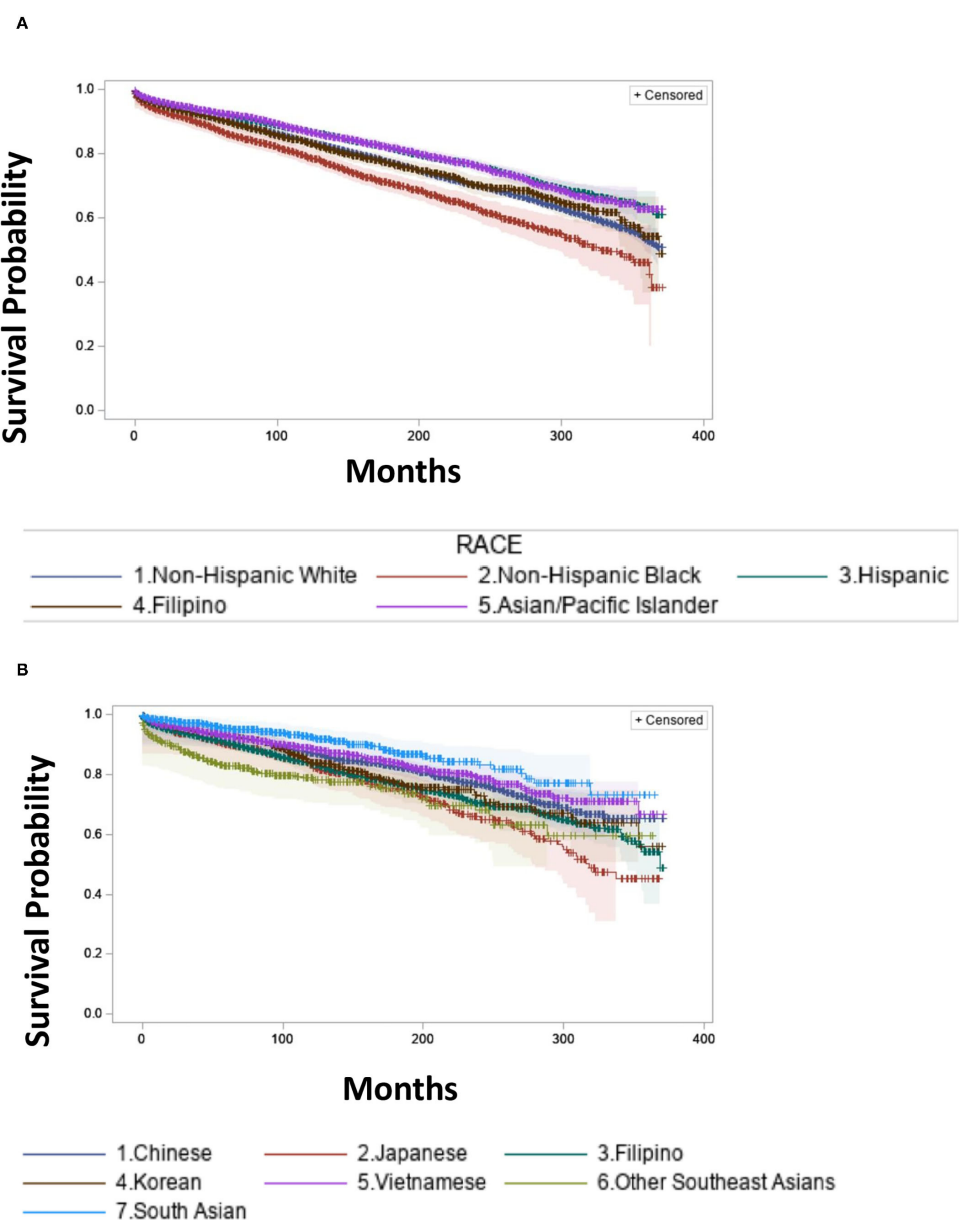
Survival estimates by race/ethnicity from 1988 to 2018 (2015 in Asian subgroups). **(A)** Survival Estimates in Non-Hispanic Whites, Non-Hispanic Black, Hispanic, Filipino, Asian/Pacific Islander excluding Filipino. **(B)** Survival Estimates in Chinese, Japanese, Filipino, Korean, Vietnamese, Other Southeast Asians, South Asian.

Overall survival for Filipinos was 91% at 5 years and 84% at 10 years, which was lower than the “all other API” group (93% at 5 years and 87% at 10 years) and Hispanics (92% at 5 years and 87% at 10 years). However, Filipinos had significantly better 5 year and 10 year OS than Non-Hispanic Blacks (87% at 5 years and 79% at 10 years) and Other Southeast Asians 83% at 5 years and 79% at 10 years (Figures 5A, B, Table 3).

**Table 3:** Thyroid cancer survival estimates by race/ethnicity.

	**1-year**			**5-year**			**10-year**		

	**SURV**	**95%CI**		**SURV**	**95%CI**		**SURV**	**95%CI**	
**Race**		**LCL**	**UCL**		**LCL**	**UCL**		**LCL**	**UCL**
**Filipino**	**95.9%**	**95.3%**	**96.4%**	**90.5%**	**89.6%**	**91.3%**	**83.6%**	**82.4%**	**84.8%**
Non-Hispanic White	96.2%	96.1%	96.4%	90.6%	90.3%	90.8%	83.8%	83.5%	84.2%
Non-Hispanic Black	94.3%	93.5%	95.0%	87.0%	85.7%	88.1%	79.2%	77.6%	80.7%
Hispanic	96.7%	96.4%	96.9%	92.1%	91.7%	92.5%	87.1%	86.6%	87.6%
Asian/Pacific Islander (A/PI) (excluding Filipinos)	96.7%	96.3%	97.0%	92.6%	92.0%	93.1%	87.0%	86.2%	87.8%
**Asian/Pacific Islander subgroups**									
Chinese	96.6%	96.0%	97.2%	92.6%	91.6%	93.5%	87.2%	85.7%	88.5%
Japanese	96.0%	94.3%	97.2%	90.1%	87.5%	92.1%	83.4%	80.1%	86.2%
Korean	97.1%	96.0%	97.9%	93.0%	91.4%	94.4%	83.9%	81.1%	86.4%
Vietnamese	96.7%	95.7%	97.5%	92.9%	91.4%	94.1%	88.6%	86.6%	90.3%
Other Southeast Asians^*^	91.2%	88.1%	93.5%	82.9%	78.7%	86.4%	78.7%	73.8%	82.7%
South Asian^**^	98.3%	97.4%	98.9%	95.3%	93.7%	96.5%	92.5%	90.1%	94.3%

Bolded values signify values of Filipino ethnicity.

### Adjusted cox hazard ratios

We showed that Filipino ethnicity compared to Non-Hispanic Whites was a significantly independent variable in our adjusted cox hazard ratio model (HR: 1.10, 95% CI 1.07–1.13, *p* < 0.0001) when adjusting for race/ethnicity, gender, age, socioeconomic status, and stage. In addition, when we looked at papillary thyroid cancer compared to Non-Hispanic Whites, Filipino ethnicity remained a significant risk factor (HR: 1.11, 95% CI 1.07–1.14, *p* < 0.0001) (Table 4). Furthermore, when stratified by Charlson score for both 0 and for 1 or greater to better delineate thyroid cancer specific risk, Filipino ethnicity was a significant independent variable for mortality (Charlson 0 HR: 1.07, 95% CI 1.02–1.11, *p* = 0.0017, Charlson 1 or greater HR: 1.07 95% CI 1.002–1.14, *p* = 0.0434) (Table 5).

**Table 4:** Multivariate hazard ratio cox regression analysis overall and in papillary thyroid cancer.

		**Overall**			**Papillary**		

		**HR**	**(95%CI)**		**HR**	**(95%CI)**	
		***n*** = **95,618**			***n*** = **82,485**		
Race	Non-Hispanic White	Reference				
	Non-Hispanic Black	1.14	1.10	1.17	1.13	1.09	1.18
	Hispanic	1.32	1.29	1.34	1.34	1.31	1.36
	**Filipino**	**1.10**	**1.07**	**1.13**	**1.11**	**1.07**	**1.14**
	Asian/Pacific Islander excluding Filipinos	1.23	1.20	1.26	1.26	1.23	1.29
Sex	Female	Reference				
	Male	1.06	1.05	1.08	1.06	1.04	1.08
Age	0–39	Reference				
	40–64	1.39	1.37	1.42	1.38	1.36	1.40
	65+	2.26	2.22	2.31	2.14	2.09	2.18
Socioeconomic status	Highest	Reference				
	Upper-middle	1.04	1.02	1.06	1.04	1.02	1.06
	Middle	1.04	1.02	1.06	1.03	1.01	1.05
	Lower-middle	1.08	1.05	1.10	1.07	1.04	1.09
	Lowest	1.10	1.07	1.12	1.09	1.06	1.11
Stage	Localized	Reference				
	Regional	1.12	1.11	1.14	1.09	1.07	1.11
	Distant	1.48	1.44	1.52	1.07	1.03	1.11
	Unknown	1.12	1.07	1.16	1.04	0.99	1.10
	*In situ*	2.03	1.72	2.41	2.64	2.14	3.25

Bolded values signify values of Filipino ethnicity.

**Table 5:** Multivariate hazard ratio cox regression analysis overall stratified by Charlson cormorbidity score.

		**Charlson 0**			**Charlson 1**+		

		**HR**	**(95%CI)**		**HR**	**(95%CI)**	
		***n*** = **55,493**			***n*** = **16,509**		
Race	Non-Hispanic White						
	Non-Hispanic Black	1.09	1.04	1.14	1.10	1.03	1.17
	Hispanic	1.24	1.21	1.27	1.14	1.09	1.18
	**Filipino**	**1.07**	**1.02**	**1.11**	**1.07**	**1.00**	**1.14**
	Asian/Pacific Islander excluding Filipinos	1.16	1.13	1.20	1.08	1.02	1.15
Sex	Female						
	Male	1.05	1.03	1.07	1.08	1.04	1.12
Age	0–39						
	40–64	1.34	1.31	1.37	1.24	1.19	1.30
	65+	2.12	2.06	2.17	1.89	1.80	1.99
Socioeconomic status	Highest						
	Upper-Middle	1.01	0.98	1.03	1.03	0.98	1.08
	Middle	1.00	0.98	1.03	1.08	1.02	1.13
	Lower-Middle	1.03	1.00	1.05	1.05	1.00	1.11
	Lowest	0.99	0.96	1.03	1.06	1.00	1.12
Stage	Localized						
	Regional	1.11	1.09	1.13	1.12	1.08	1.16
	Distant	1.38	1.33	1.43	1.98	1.88	2.09
	Unknown	1.31	1.21	1.42	1.73	1.55	1.92
	*In situ*	1.56	1.15	2.13	3.93	2.48	6.24

Bolded values signify values of Filipino ethnicity.

## Discussion

Our study highlights some of the population-based differences in Filipino thyroid cancer cases in California compared to other race/ethnicities and our analysis highlights that Filipino ethnicity is a significant risk factor in all-cause mortality in thyroid cancer. Filipinos had higher incidences of T4 tumor status, N1 nodal status, lymphovascular invasion, and multifocality compared to Non-Hispanic Whites, Non-Hispanic Blacks, and some of the Asian subgroups. We then showed that Filipinos have the highest AAMR. When adjusting for sex, age at diagnosis, socioeconomic status, staging, and stratifying by comorbidity score, Filipino ethnicity remained a significant independent variable for mortality risk in thyroid cancer.

As we see these differences beyond a local level, we should re-consider some of our previous hypotheses for these persistent differences in Filipinos compared to other ethnicities. To start, contrary to previous thoughts showing Filipinos having higher incidence of early-stage thyroid cancer due to subsequent over diagnosis is not true, as there is less early diagnosis and a higher likelihood of Stage IV thyroid cancer at diagnosis among Filipinos compared with Non-Hispanic Whites, Non-Hispanic Blacks, Hispanics and nearly all Asian subgroups (16). This is further supported by a recent study showing that incidence rates of small tumors (< 2 cm) increased in non-Filipino Asians and Non-Hispanic Whites but it did not in Filipinos suggesting that much of the increases in Filipino incidences are more likely due to advanced thyroid cancer presentations (7).

Our findings showing increased prevalence of key high-risk features notably Stage IV at diagnosis at a population level highlight the need to evaluate whether molecular alterations play a role in more severe presentations of thyroid cancer in Filipinos. BRAF mutations have been shown to be in about 45% papillary thyroid cancer cases (17). In 2014, a study in Korea showed that presence of BRAF V600E alleles had a significant association with extrathyroidal extension, absence of chronic lymphocytic thyroiditis, and increased tumor size (18). Other studies of Asian populations have shown varying rates of BRAF mutations ranging from 29 to 79% (19–25). Meanwhile, a retrospective study in Hawaii in 2011 showed a high incidence of BRAF mutations (83.8%) among the Filipino population (26). A recent retrospective study of 64 sequential patients in the Philippines who underwent thyroidectomy in 2016 showed that 12/17 patients who had papillary thyroid carcinoma harbored a BRAF V600E mutation with extrathyroidal extension in 7/18 patients, multifocality in 6/18 patients, and lymph node involvement in 8/18 patients (27). However, among native born patients in the Philippines, the reported incidence of BRAF alterations was lower overall at 38.46% in a single center compared to other referenced Japanese, Chinese, Taiwanese, and Korean populations (20–25, 28). Moreover, both somatic and germline alterations need to be better elucidated. We showed that Filipinos for example were less likely to have medullary thyroid carcinoma, which can be associated with the RET proto-oncogene and Multiple Endocrine Neoplasia 2A and 2B, than Non-Hispanic Whites, Non-Hispanic Blacks, Hispanics, and other Asian subgroups specifically Chinese and South Asians (29). As patients with BRAF V600E, NTRK, and RET mutations now have targeted therapy for metastatic thyroid cancer, further larger cohort prospective studies on molecular profiling could elucidate differences in thyroid cancer in Filipinos compared to other race/ethnic groups and to also see if they could explain that possible increased prevalence of features such as larger tumor sizes, lymph node involvement, and multifocality may be tied to increased incidence and mortality risks.

Disaggregation of Asian subgroups allowed observation of notable differences amongst Filipinos and other Asian subgroups. These differences are most stark amongst Filipinos and Chinese. Filipinos were shown to have greater likelihood of Stage IV thyroid cancer and other high-risk features. Meanwhile, Filipino patients were less likely to have medullary thyroid cancer cases. Overall, Filipinos were shown to have worse 5 year and 10 year OS compared to Chinese patients. These differences are not limited to solely Chinese and Filipinos, as we also see between Filipinos and Vietnamese that Filipinos were less likely than Vietnamese to have follicular thyroid cancer and more likely to have T4 tumor status and multifocality. While notable differences in patterns of cancer have been shown between Filipinos and other Asian subgroups, little has been done to highlight the possible genetic differences (30). Our findings show the need to do studies comparing key mutations in thyroid cancer such as BRAF and RET between Filipinos and other Asian subgroups to see if they may explain differences in clinical characteristics of thyroid cancer.

The strength of this study is being able to demonstrate at a cancer registry level that Filipinos have significant incidence of multiple high-risk features of increased tumor size, extrathyroidal extension, and Stage IV disease relative to other race/ethnicities and while demonstrating an increased AAMR. Furthermore, when stratifying for having other comorbidities, Filipino ethnicity remained a significantly independent variable for mortality risk. Another important study strength is the large sample size of Filipinos in CCR data; Filipinos in California constitute nearly 1.6 million of the 4 million Filipinos in the United States so our study reflects a sizeable portion of thyroid cancers occurring in the U.S. Filipino population (31). Limitations to this study include the fact that much of the data for race is self-reported and given that CCR only reports 1 racial or ethnic group per patient, racial designations may not be entirely accurate. We were limited by small sample sizes to include American Indian and Hawaiian/Pacific Islanders or to disaggregate between Hispanic Black and Hispanic Asian in our analysis. Additionally, our data ranges from 1988 to 2018, and therefore during this time there were multiple changes in the AJCC TNM staging system for thyroid cancer; some of tumor and nodal specific staging may be different during this time. Akin to other registry databases, there was missing data, primarily around 5% of the patients with the notable exception of multifocality data, which had about 35% missing. For missing data, we excluded those subjects for the respective analysis we were doing. No information on mutations (i.e., BRAF or RET) was available. Ultimately, our study shows that on a population level that Filipinos have increased prevalence of high-risk pathological features in advanced thyroid cancer and increased mortality risk. Future studies will need to focus on biological and socioeconomic analyses to better understand the connection between the increase incidence of these high-risk pathological features and mortality risk.

## Conclusion

Our analysis of Filipinos using the California Cancer Registry demonstrated that Filipinos had the higher incidence of multiple important pathologic findings in advanced thyroid cancer and the highest AAMR in 2015 among all race/ethnicities and that Filipino ethnicity is an independently significant variable in mortality risk. These findings warrant further research into understanding the connection between these higher incidences and increased mortality risk.

## Data availability statement

The original contributions presented in the study are included in the article/Supplementary material, further inquiries can be directed to the corresponding author.

## Ethics statement

Ethical review and approval was not required for the study on human participants in accordance with the local legislation and institutional requirements. Written informed consent for participation was not required for this study in accordance with the national legislation and the institutional requirements.

## Author contributions

RH: conceptualization, investigation, supervision, project administration, data curation, methodology, formal analysis, visualization, writing—original draft preparation, and writing—review and editing. K-YT: data curation, methodology, formal analysis, and writing—review and editing. KC: investigation, visualization, writing—original draft preparation, and writing—review and editing. KW and AL: writing—review and editing. JN: investigation, supervision, writing—original draft preparation, and writing—review and editing. LL: conceptualization, investigation, supervision, project administration, formal analysis, writing—original draft preparation, and writing—review and editing. All authors contributed to the article and approved the submitted version.
