# Supplementary material for: Characterization of mortality and high-risk characteristics of thyroid cancer in Filipinos using the California Cancer Registry
Source: Front Public Health. 2023 Jan 19;10:1104607. doi: 10.3389/fpubh.2022.1104607 (PMC9893642; doi:10.3389/fpubh.2022.1104607)
Supplement: Supplementary file 1 [file Presentation_1.PPTX]

## Slide 1
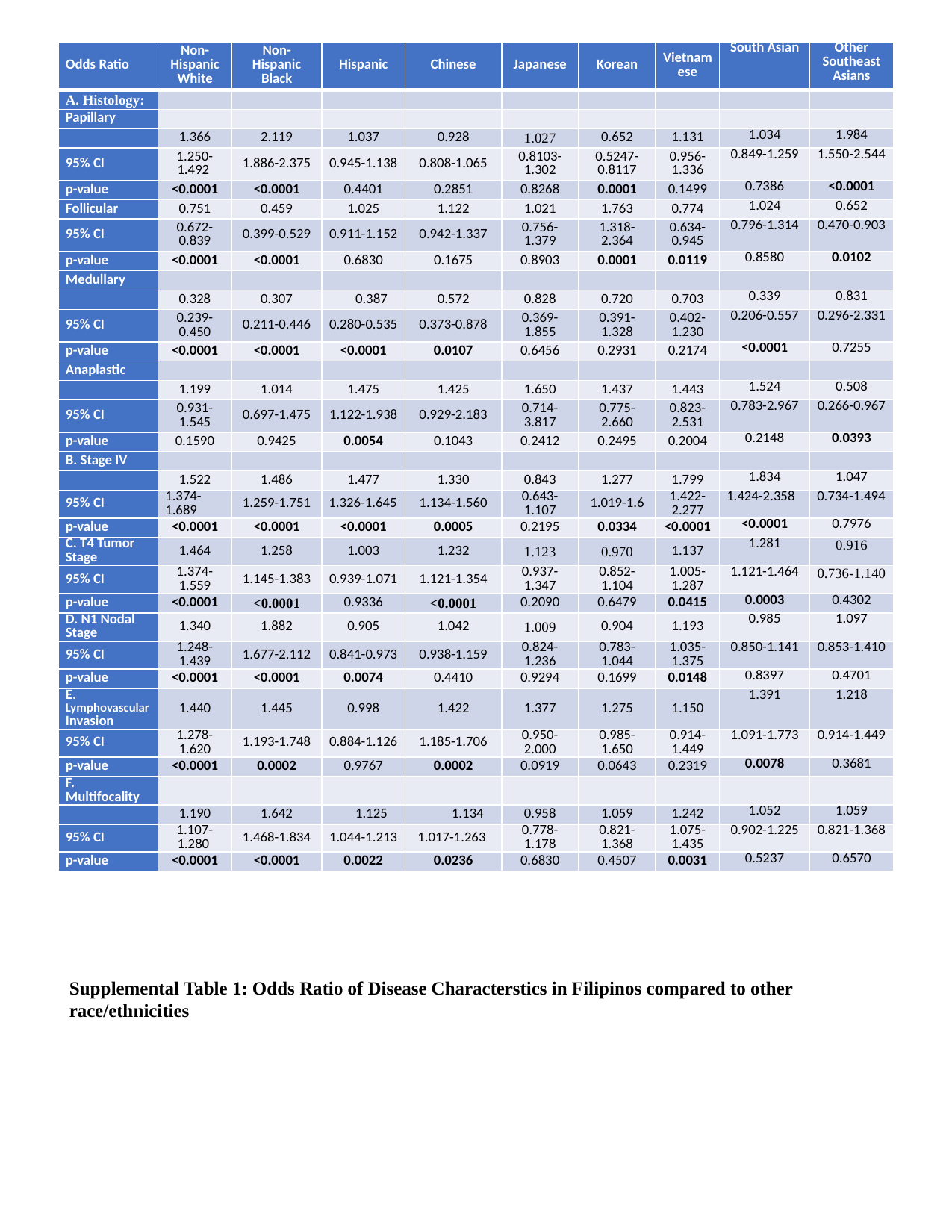

| Odds Ratio | Non-Hispanic White | Non-Hispanic Black | Hispanic | Chinese | Japanese | Korean | Vietnamese | South Asian | Other Southeast Asians |
| --- | --- | --- | --- | --- | --- | --- | --- | --- | --- |
| A. Histology: | | | | | | | | | |
| Papillary | | | | | | | | | |
| | 1.366 | 2.119 | 1.037 | 0.928 | 1.027 | 0.652 | 1.131 | 1.034 | 1.984 |
| 95% CI | 1.250-1.492 | 1.886-2.375 | 0.945-1.138 | 0.808-1.065 | 0.8103-1.302 | 0.5247-0.8117 | 0.956-1.336 | 0.849-1.259 | 1.550-2.544 |
| p-value | <0.0001 | <0.0001 | 0.4401 | 0.2851 | 0.8268 | 0.0001 | 0.1499 | 0.7386 | <0.0001 |
| Follicular | 0.751 | 0.459 | 1.025 | 1.122 | 1.021 | 1.763 | 0.774 | 1.024 | 0.652 |
| 95% CI | 0.672-0.839 | 0.399-0.529 | 0.911-1.152 | 0.942-1.337 | 0.756-1.379 | 1.318-2.364 | 0.634-0.945 | 0.796-1.314 | 0.470-0.903 |
| p-value | <0.0001 | <0.0001 | 0.6830 | 0.1675 | 0.8903 | 0.0001 | 0.0119 | 0.8580 | 0.0102 |
| Medullary | | | | | | | | | |
| | 0.328 | 0.307 | 0.387 | 0.572 | 0.828 | 0.720 | 0.703 | 0.339 | 0.831 |
| 95% CI | 0.239-0.450 | 0.211-0.446 | 0.280-0.535 | 0.373-0.878 | 0.369-1.855 | 0.391-1.328 | 0.402-1.230 | 0.206-0.557 | 0.296-2.331 |
| p-value | <0.0001 | <0.0001 | <0.0001 | 0.0107 | 0.6456 | 0.2931 | 0.2174 | <0.0001 | 0.7255 |
| Anaplastic | | | | | | | | | |
| | 1.199 | 1.014 | 1.475 | 1.425 | 1.650 | 1.437 | 1.443 | 1.524 | 0.508 |
| 95% CI | 0.931-1.545 | 0.697-1.475 | 1.122-1.938 | 0.929-2.183 | 0.714-3.817 | 0.775-2.660 | 0.823-2.531 | 0.783-2.967 | 0.266-0.967 |
| p-value | 0.1590 | 0.9425 | 0.0054 | 0.1043 | 0.2412 | 0.2495 | 0.2004 | 0.2148 | 0.0393 |
| B. Stage IV | | | | | | | | | |
| | 1.522 | 1.486 | 1.477 | 1.330 | 0.843 | 1.277 | 1.799 | 1.834 | 1.047 |
| 95% CI | 1.374-1.689 | 1.259-1.751 | 1.326-1.645 | 1.134-1.560 | 0.643-1.107 | 1.019-1.6 | 1.422-2.277 | 1.424-2.358 | 0.734-1.494 |
| p-value | <0.0001 | <0.0001 | <0.0001 | 0.0005 | 0.2195 | 0.0334 | <0.0001 | <0.0001 | 0.7976 |
| C. T4 Tumor Stage | 1.464 | 1.258 | 1.003 | 1.232 | 1.123 | 0.970 | 1.137 | 1.281 | 0.916 |
| 95% CI | 1.374-1.559 | 1.145-1.383 | 0.939-1.071 | 1.121-1.354 | 0.937-1.347 | 0.852-1.104 | 1.005-1.287 | 1.121-1.464 | 0.736-1.140 |
| p-value | <0.0001 | <0.0001 | 0.9336 | <0.0001 | 0.2090 | 0.6479 | 0.0415 | 0.0003 | 0.4302 |
| D. N1 Nodal Stage | 1.340 | 1.882 | 0.905 | 1.042 | 1.009 | 0.904 | 1.193 | 0.985 | 1.097 |
| 95% CI | 1.248-1.439 | 1.677-2.112 | 0.841-0.973 | 0.938-1.159 | 0.824-1.236 | 0.783-1.044 | 1.035-1.375 | 0.850-1.141 | 0.853-1.410 |
| p-value | <0.0001 | <0.0001 | 0.0074 | 0.4410 | 0.9294 | 0.1699 | 0.0148 | 0.8397 | 0.4701 |
| E. Lymphovascular Invasion | 1.440 | 1.445 | 0.998 | 1.422 | 1.377 | 1.275 | 1.150 | 1.391 | 1.218 |
| 95% CI | 1.278-1.620 | 1.193-1.748 | 0.884-1.126 | 1.185-1.706 | 0.950-2.000 | 0.985-1.650 | 0.914-1.449 | 1.091-1.773 | 0.914-1.449 |
| p-value | <0.0001 | 0.0002 | 0.9767 | 0.0002 | 0.0919 | 0.0643 | 0.2319 | 0.0078 | 0.3681 |
| F. Multifocality | | | | | | | | | |
| | 1.190 | 1.642 | 1.125 | 1.134 | 0.958 | 1.059 | 1.242 | 1.052 | 1.059 |
| 95% CI | 1.107-1.280 | 1.468-1.834 | 1.044-1.213 | 1.017-1.263 | 0.778-1.178 | 0.821-1.368 | 1.075-1.435 | 0.902-1.225 | 0.821-1.368 |
| p-value | <0.0001 | <0.0001 | 0.0022 | 0.0236 | 0.6830 | 0.4507 | 0.0031 | 0.5237 | 0.6570 |
Supplemental Table 1: Odds Ratio of Disease Characterstics in Filipinos compared to other race/ethnicities

## Slide 2
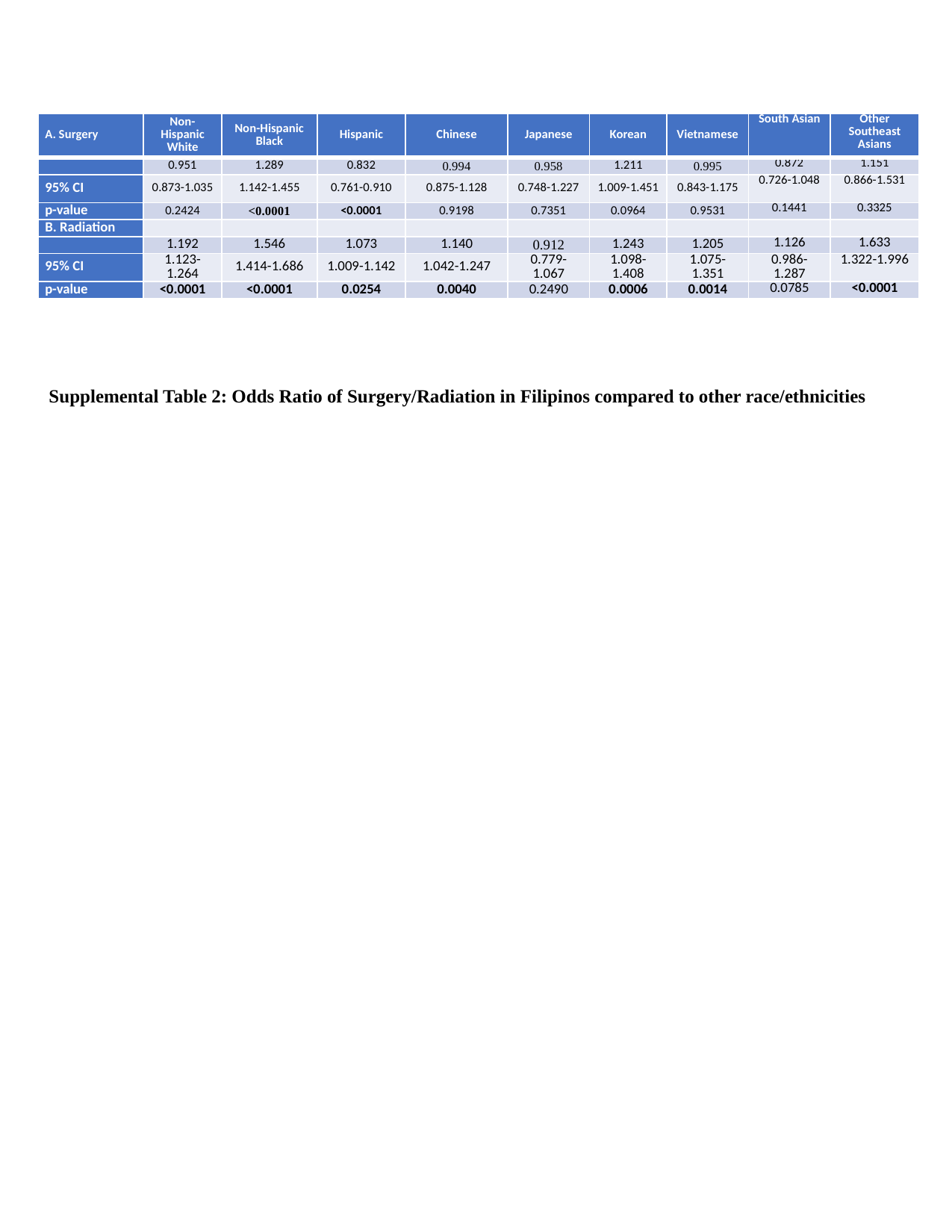

| A. Surgery | Non-Hispanic White | Non-Hispanic Black | Hispanic | Chinese | Japanese | Korean | Vietnamese | South Asian | Other Southeast Asians |
| --- | --- | --- | --- | --- | --- | --- | --- | --- | --- |
| | 0.951 | 1.289 | 0.832 | 0.994 | 0.958 | 1.211 | 0.995 | 0.872 | 1.151 |
| 95% CI | 0.873-1.035 | 1.142-1.455 | 0.761-0.910 | 0.875-1.128 | 0.748-1.227 | 1.009-1.451 | 0.843-1.175 | 0.726-1.048 | 0.866-1.531 |
| p-value | 0.2424 | <0.0001 | <0.0001 | 0.9198 | 0.7351 | 0.0964 | 0.9531 | 0.1441 | 0.3325 |
| B. Radiation | | | | | | | | | |
| | 1.192 | 1.546 | 1.073 | 1.140 | 0.912 | 1.243 | 1.205 | 1.126 | 1.633 |
| 95% CI | 1.123-1.264 | 1.414-1.686 | 1.009-1.142 | 1.042-1.247 | 0.779-1.067 | 1.098-1.408 | 1.075-1.351 | 0.986-1.287 | 1.322-1.996 |
| p-value | <0.0001 | <0.0001 | 0.0254 | 0.0040 | 0.2490 | 0.0006 | 0.0014 | 0.0785 | <0.0001 |
Supplemental Table 2: Odds Ratio of Surgery/Radiation in Filipinos compared to other race/ethnicities

## Slide 3
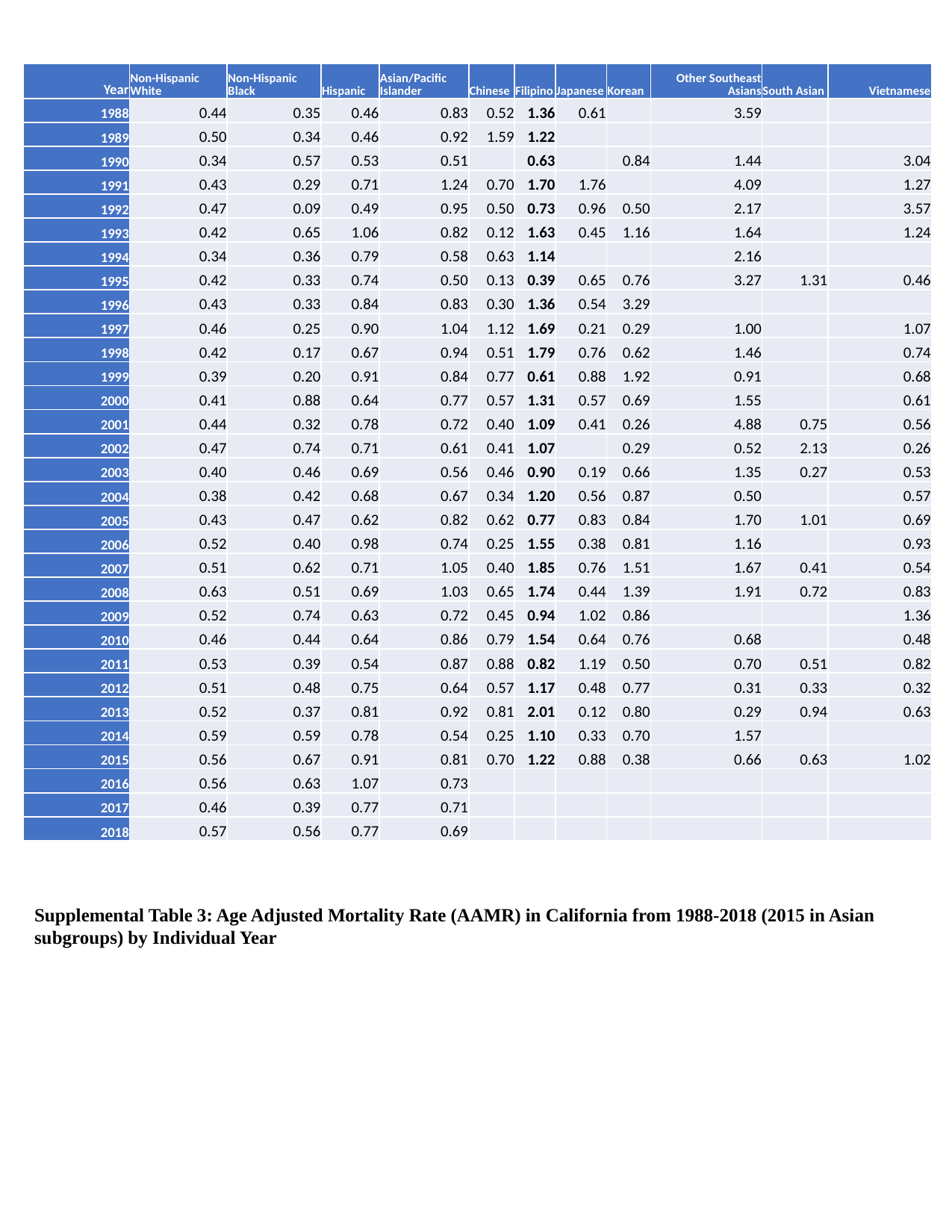

| Year | Non-Hispanic White | Non-Hispanic Black | Hispanic | Asian/Pacific Islander | Chinese | Filipino | Japanese | Korean | Other Southeast Asians | South Asian | Vietnamese |
| --- | --- | --- | --- | --- | --- | --- | --- | --- | --- | --- | --- |
| 1988 | 0.44 | 0.35 | 0.46 | 0.83 | 0.52 | 1.36 | 0.61 | | 3.59 | | |
| 1989 | 0.50 | 0.34 | 0.46 | 0.92 | 1.59 | 1.22 | | | | | |
| 1990 | 0.34 | 0.57 | 0.53 | 0.51 | | 0.63 | | 0.84 | 1.44 | | 3.04 |
| 1991 | 0.43 | 0.29 | 0.71 | 1.24 | 0.70 | 1.70 | 1.76 | | 4.09 | | 1.27 |
| 1992 | 0.47 | 0.09 | 0.49 | 0.95 | 0.50 | 0.73 | 0.96 | 0.50 | 2.17 | | 3.57 |
| 1993 | 0.42 | 0.65 | 1.06 | 0.82 | 0.12 | 1.63 | 0.45 | 1.16 | 1.64 | | 1.24 |
| 1994 | 0.34 | 0.36 | 0.79 | 0.58 | 0.63 | 1.14 | | | 2.16 | | |
| 1995 | 0.42 | 0.33 | 0.74 | 0.50 | 0.13 | 0.39 | 0.65 | 0.76 | 3.27 | 1.31 | 0.46 |
| 1996 | 0.43 | 0.33 | 0.84 | 0.83 | 0.30 | 1.36 | 0.54 | 3.29 | | | |
| 1997 | 0.46 | 0.25 | 0.90 | 1.04 | 1.12 | 1.69 | 0.21 | 0.29 | 1.00 | | 1.07 |
| 1998 | 0.42 | 0.17 | 0.67 | 0.94 | 0.51 | 1.79 | 0.76 | 0.62 | 1.46 | | 0.74 |
| 1999 | 0.39 | 0.20 | 0.91 | 0.84 | 0.77 | 0.61 | 0.88 | 1.92 | 0.91 | | 0.68 |
| 2000 | 0.41 | 0.88 | 0.64 | 0.77 | 0.57 | 1.31 | 0.57 | 0.69 | 1.55 | | 0.61 |
| 2001 | 0.44 | 0.32 | 0.78 | 0.72 | 0.40 | 1.09 | 0.41 | 0.26 | 4.88 | 0.75 | 0.56 |
| 2002 | 0.47 | 0.74 | 0.71 | 0.61 | 0.41 | 1.07 | | 0.29 | 0.52 | 2.13 | 0.26 |
| 2003 | 0.40 | 0.46 | 0.69 | 0.56 | 0.46 | 0.90 | 0.19 | 0.66 | 1.35 | 0.27 | 0.53 |
| 2004 | 0.38 | 0.42 | 0.68 | 0.67 | 0.34 | 1.20 | 0.56 | 0.87 | 0.50 | | 0.57 |
| 2005 | 0.43 | 0.47 | 0.62 | 0.82 | 0.62 | 0.77 | 0.83 | 0.84 | 1.70 | 1.01 | 0.69 |
| 2006 | 0.52 | 0.40 | 0.98 | 0.74 | 0.25 | 1.55 | 0.38 | 0.81 | 1.16 | | 0.93 |
| 2007 | 0.51 | 0.62 | 0.71 | 1.05 | 0.40 | 1.85 | 0.76 | 1.51 | 1.67 | 0.41 | 0.54 |
| 2008 | 0.63 | 0.51 | 0.69 | 1.03 | 0.65 | 1.74 | 0.44 | 1.39 | 1.91 | 0.72 | 0.83 |
| 2009 | 0.52 | 0.74 | 0.63 | 0.72 | 0.45 | 0.94 | 1.02 | 0.86 | | | 1.36 |
| 2010 | 0.46 | 0.44 | 0.64 | 0.86 | 0.79 | 1.54 | 0.64 | 0.76 | 0.68 | | 0.48 |
| 2011 | 0.53 | 0.39 | 0.54 | 0.87 | 0.88 | 0.82 | 1.19 | 0.50 | 0.70 | 0.51 | 0.82 |
| 2012 | 0.51 | 0.48 | 0.75 | 0.64 | 0.57 | 1.17 | 0.48 | 0.77 | 0.31 | 0.33 | 0.32 |
| 2013 | 0.52 | 0.37 | 0.81 | 0.92 | 0.81 | 2.01 | 0.12 | 0.80 | 0.29 | 0.94 | 0.63 |
| 2014 | 0.59 | 0.59 | 0.78 | 0.54 | 0.25 | 1.10 | 0.33 | 0.70 | 1.57 | | |
| 2015 | 0.56 | 0.67 | 0.91 | 0.81 | 0.70 | 1.22 | 0.88 | 0.38 | 0.66 | 0.63 | 1.02 |
| 2016 | 0.56 | 0.63 | 1.07 | 0.73 | | | | | | | |
| 2017 | 0.46 | 0.39 | 0.77 | 0.71 | | | | | | | |
| 2018 | 0.57 | 0.56 | 0.77 | 0.69 | | | | | | | |
Supplemental Table 3: Age Adjusted Mortality Rate (AAMR) in California from 1988-2018 (2015 in Asian subgroups) by Individual Year

## Slide 4
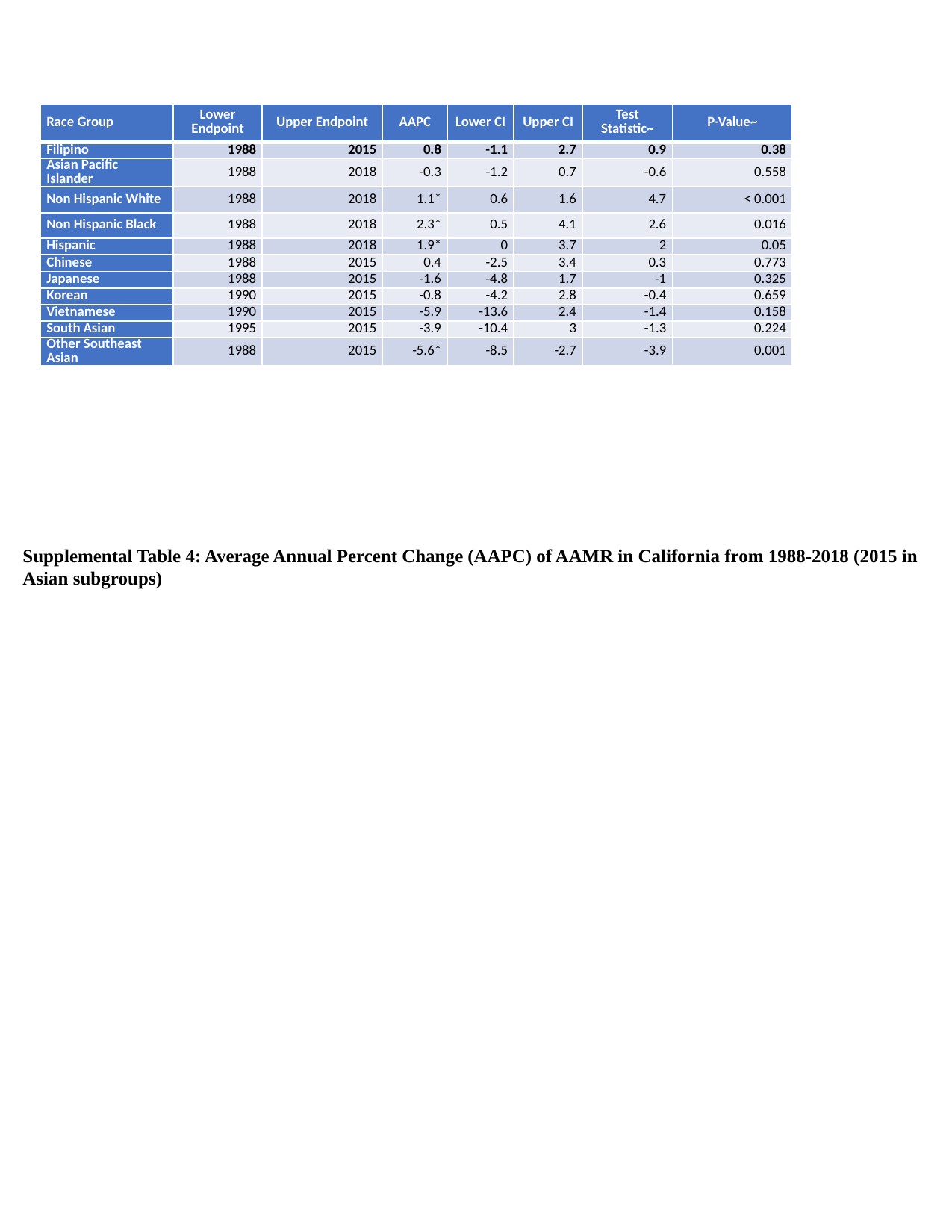

| Race Group | Lower Endpoint | Upper Endpoint | AAPC | Lower CI | Upper CI | Test Statistic~ | P-Value~ |
| --- | --- | --- | --- | --- | --- | --- | --- |
| Filipino | 1988 | 2015 | 0.8 | -1.1 | 2.7 | 0.9 | 0.38 |
| Asian Pacific Islander | 1988 | 2018 | -0.3 | -1.2 | 0.7 | -0.6 | 0.558 |
| Non Hispanic White | 1988 | 2018 | 1.1\* | 0.6 | 1.6 | 4.7 | < 0.001 |
| Non Hispanic Black | 1988 | 2018 | 2.3\* | 0.5 | 4.1 | 2.6 | 0.016 |
| Hispanic | 1988 | 2018 | 1.9\* | 0 | 3.7 | 2 | 0.05 |
| Chinese | 1988 | 2015 | 0.4 | -2.5 | 3.4 | 0.3 | 0.773 |
| Japanese | 1988 | 2015 | -1.6 | -4.8 | 1.7 | -1 | 0.325 |
| Korean | 1990 | 2015 | -0.8 | -4.2 | 2.8 | -0.4 | 0.659 |
| Vietnamese | 1990 | 2015 | -5.9 | -13.6 | 2.4 | -1.4 | 0.158 |
| South Asian | 1995 | 2015 | -3.9 | -10.4 | 3 | -1.3 | 0.224 |
| Other Southeast Asian | 1988 | 2015 | -5.6\* | -8.5 | -2.7 | -3.9 | 0.001 |
Supplemental Table 4: Average Annual Percent Change (AAPC) of AAMR in California from 1988-2018 (2015 in Asian subgroups)
